# Induction and measurement of the early stage of a host‐parasite interaction using a combined optical trapping and Raman microspectroscopy system

**DOI:** 10.1002/jbio.201960065

**Published:** 2019-11-28

**Authors:** Faris Sinjab, Hany M. Elsheikha, Max Dooley, Ioan Notingher

**Affiliations:** ^1^ School of Physics and Astronomy, University Park Nottingham University of Nottingham Nottingham UK; ^2^ Faculty of Medicine and Health Sciences, School of Veterinary Medicine and Science University of Nottingham Loughborough UK

**Keywords:** blood‐brain‐barrier, host‐parasite interaction, optical trapping, Raman spectroscopy, *Toxoplasma gondii*

## Abstract

Understanding and quantifying the temporal acquisition of host cell molecules by intracellular pathogens is fundamentally important in biology. In this study, a recently developed holographic optical trapping (HOT)‐based Raman microspectroscopy (RMS) instrument is applied to detect, characterize and monitor in real time the molecular trafficking of a specific molecular species (isotope‐labeled phenylalanine (L‐Phe(D8)) at the single cell level. This approach enables simultaneous measurement of the chemical composition of human cerebrovascular endothelial cells and the protozoan parasite *Toxoplasma gondii* in isolation at the very start of the infection process. Using a model to decouple measurement contributions from host and pathogen sampling in the excitation volume, the data indicate that manipulating parasites with HOT coupled with RMS chemical readout was an effective method for measurement of L‐Phe(D8) transfer from host cells to parasites in real‐time, from the moment the parasite enters the host cell.
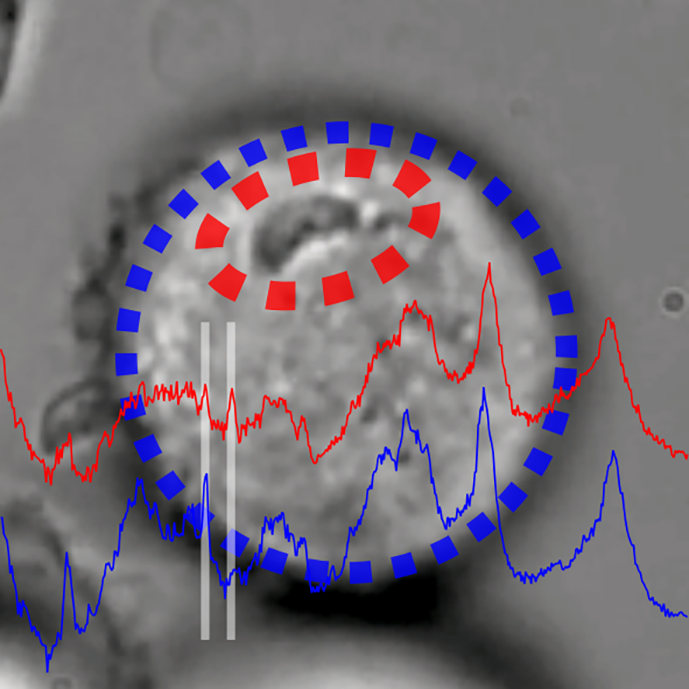

## Graphical abstract

Understanding and quantifying the temporal acquisition of host cell molecules by intracellular pathogens is fundamentally important in biology. In this study, a recently developed holographic optical trapping (HOT)‐based Raman microspectroscopy (RMS) instrument is applied to detect, characterize and monitor in real time the molecular trafficking of a specific molecular species at the single cell level. This approach enables simultaneous measurement of the chemical composition of human cerebrovascular endothelial cells and the protozoan parasite *Toxoplasma gondii* in isolation at the very start of the infection process.

**Abbreviations**
BBB: blood‐brain‐barrier
DMD: digital micromirror device
EC: endothelial cell
HOT: holographic optical trapping
LC‐SLM: liquid‐crystal spatial light modulator
PSF: point spread function
RMS: Raman microspectroscopy

## 1 INTRODUCTION

All biological processes involve highly orchestrated interactions between cells at a molecular level. Understanding these complex processes requires the ability to manipulate cells and control their interactions without compromising their viability or functional properties. At the same time, tools are needed to measure the underlying molecular properties of cells to detect changes in their biochemical properties (eg, transfer of molecules involved in cell‐cell signaling). In broad terms, the research problem addressed herein is how molecular trafficking events can be measured from the moment the parasite first encounters its surrogate host cell.

The protozoan *Toxoplasma gondii* is a frequent cause of brain infection and can cause parasitic encephalitis, a life‐threatening infection, predominately found in immunocompromised individuals.^1^ As *T. gondii* has the ability to invade the brain, there is still a dire need to better understand the mechanisms by which this parasite crosses the blood‐brain‐barrier (BBB) and cause brain damage. The strict obligate lifestyle of *T. gondii* means that this parasite must have developed elaborate mechanisms to acquire specific nutrients at specific times during its intracellular developmental cycle to sustain its own growth and establishment of infection.^2^ Therefore, understanding the metabolic and chemical alterations that accompany *T. gondii* infection has gained significant attention in the last few years.^3^, ^4^, ^5^, ^6^, ^7^

Raman microspectroscopy (RMS) allows sensitive and specific measurement of live cells, whereby vibrational spectra from diffraction‐limited sampling volumes can be measured which indicate the presence of endogenous macromolecules, such as proteins, lipids and nucleic acids in cells.^8^ RMS has been used to study the interaction of parasites and host cells, such as *Neospora caninum* 9 and *T. gondii*.^10^ Additionally, stable isotope labeling in culture (SILAC) was used to increase the molecular specificity of the Raman spectroscopy measurements, allowing monitoring the exchange of stable isotope‐labeled phenylalanine (_L_‐Phe(D8)) between human retinal pigment epithelial cells (ARPE‐19) and *T. gondii* 11 or *Acanthamoeba castellanii*.^12^ Although RMS provides spatial and temporal details of the biological molecule exchange between the parasite and its host cell, it provides no control over this interaction (ie, cells come in contact by chance), making it difficult to investigate the very early stages (first tens of minutes) in the host‐parasite interaction.

Here, we aimed to overcome these limitations by combining the high resolution RMS with custom‐built holographic optical tweezers (HOT) to study the dynamic behavior of host‐parasite interaction and to characterize in real‐time the molecule trafficking between host cell and the parasite in action. Optical trapping can be used to manipulate and induce host‐parasite interactions using light.^13^, ^14^, ^15^, ^16^ By enabling control over the cell‐cell interaction, optical trapping has enabled investigation of the early events in intercellular biological processes, such as phagocytosis and synapse formation.^17^ Integration of HOT with RMS can be achieved by using the laser traps as an excitation source for Raman spectroscopy, allowing the measurement of the molecular properties of the optically trapped cells.^18^ Recently, an integrated HOT and RMS system was demonstrated to be powerful for measurement and manipulation of live cells and organisms.^19^, ^20^ HOT‐RMS has potential for studying interaction between parasites and host cells noninvasively, and observing the molecular specific changes of isotope labels from the very earliest stages.

The application of HOT‐RMS has expanded in the present study to monitor the uptake of a single molecule (_L_‐Phe(D8)) by intracellular parasite *T. gondii* from the surrogate human cerebrovascular endothelial cells (ECs) in real time by inducing the host‐parasite interaction using optical tweezers and measuring shifts in Raman spectra that accompany changes in the composition of Phe in both host cell and the parasite. This is important to enable new fundamental information about dynamic trafficking of chemical molecules between parasite and its host cell in a direct and controlled manner.

## 2 METHODS

### 2.1 HOT‐RMS instrument

**Figure 1:**
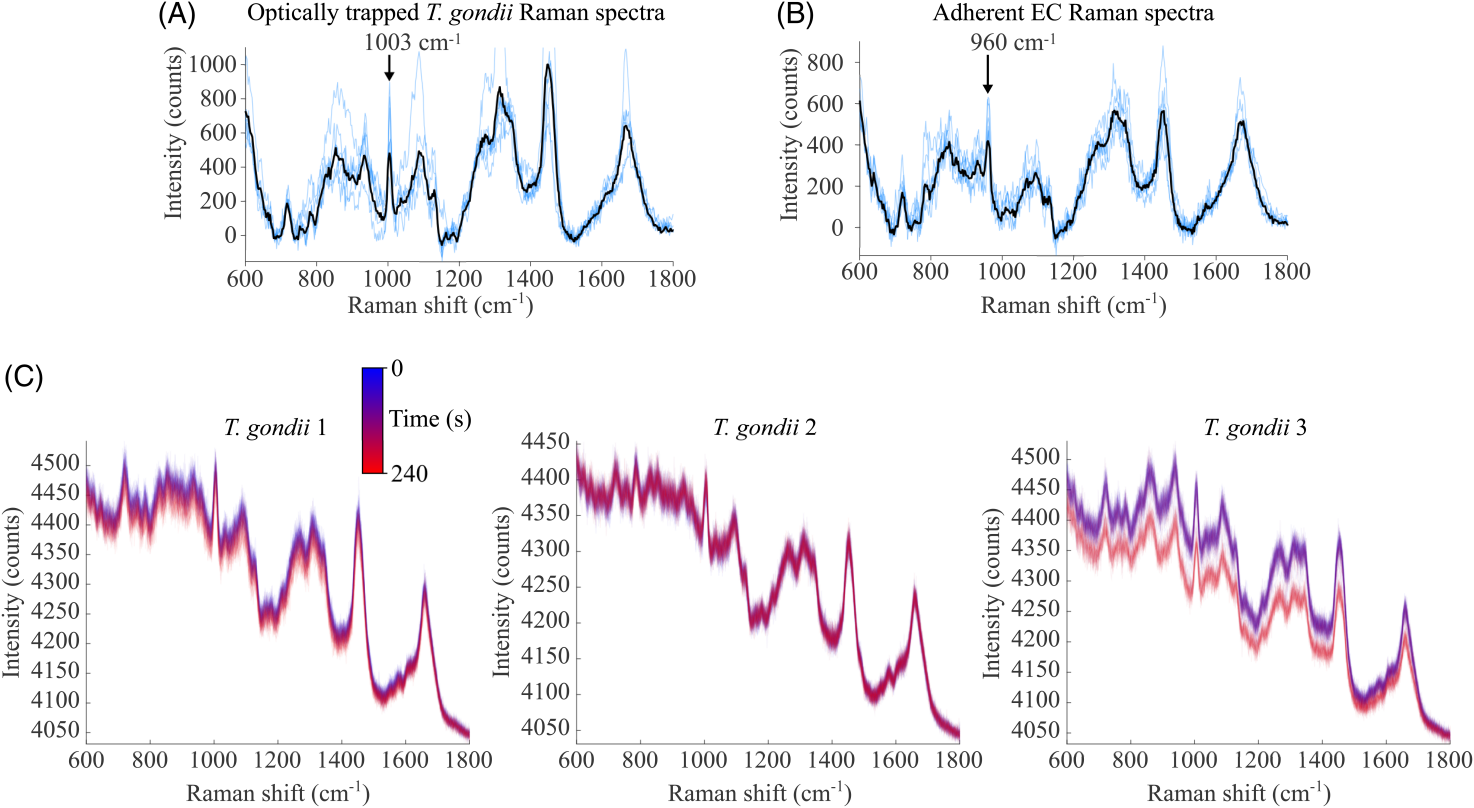
Raman spectra of (A) trapped *T. gondii* tachyzoites (blue: six separate optically trapped *T. gondii* tachyzoites, black denotes the mean) and (B) adherent endothelial cells grown in medium with _L_‐Phe(D8) (blue: n = 6 locations from the cytoplasm of single EC, black denotes the mean). (C) Examples of time‐course Raman spectra (raw data) for three different individually trapped *T. gondii* tachyzoites over 240 seconds. Acquisition time for individual spectra was 2 seconds

**Figure 2:**
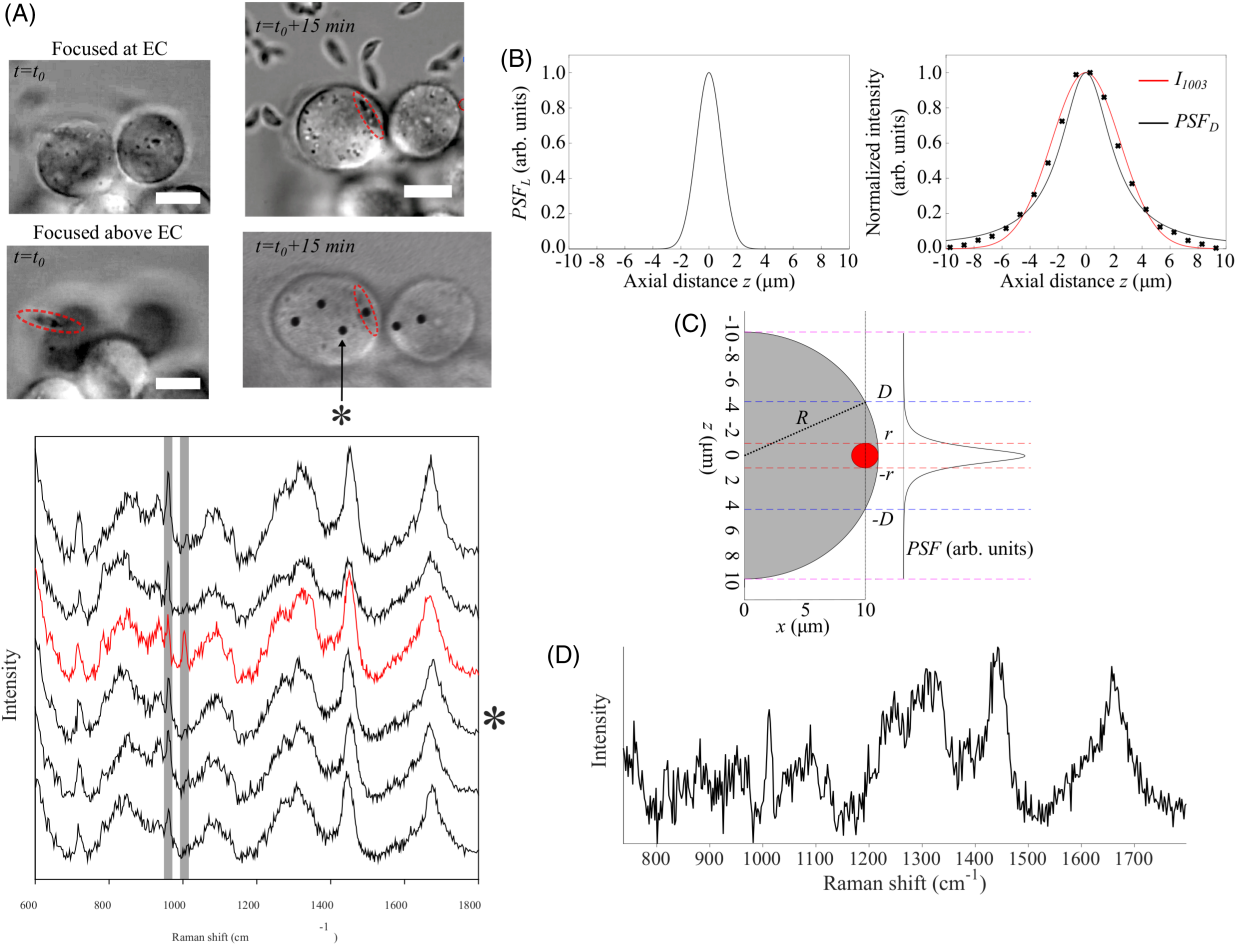
(A) Brightfield images of host endothelial cells infected by *T. gondii* (scale bar 10 μm) and Raman spectra from the EC cytoplasm (black) and at the position where the *T. gondii* was located (outlined by dashed red line). The * indicates the spectrum *S*_EC_ (and acquisition location), used for subtraction (The six Raman spectra were acquired in parallel in 30 seconds). (B) The point spread functions for laser excitation (left) and confocal Raman detection (right). (C) the simplified model of the *T. gondii* (red) and EC (gray) and the calculated overall point spread function of the instrument PSF(*z*) = PSF_*L*_(*z*) × PSF_*D*_(*z*). (D) The calculated Raman spectrum of *T. gondii*, *S*_TG_, after subtracting the contribution of the host cell *S*_EC_

**Figure 3:**
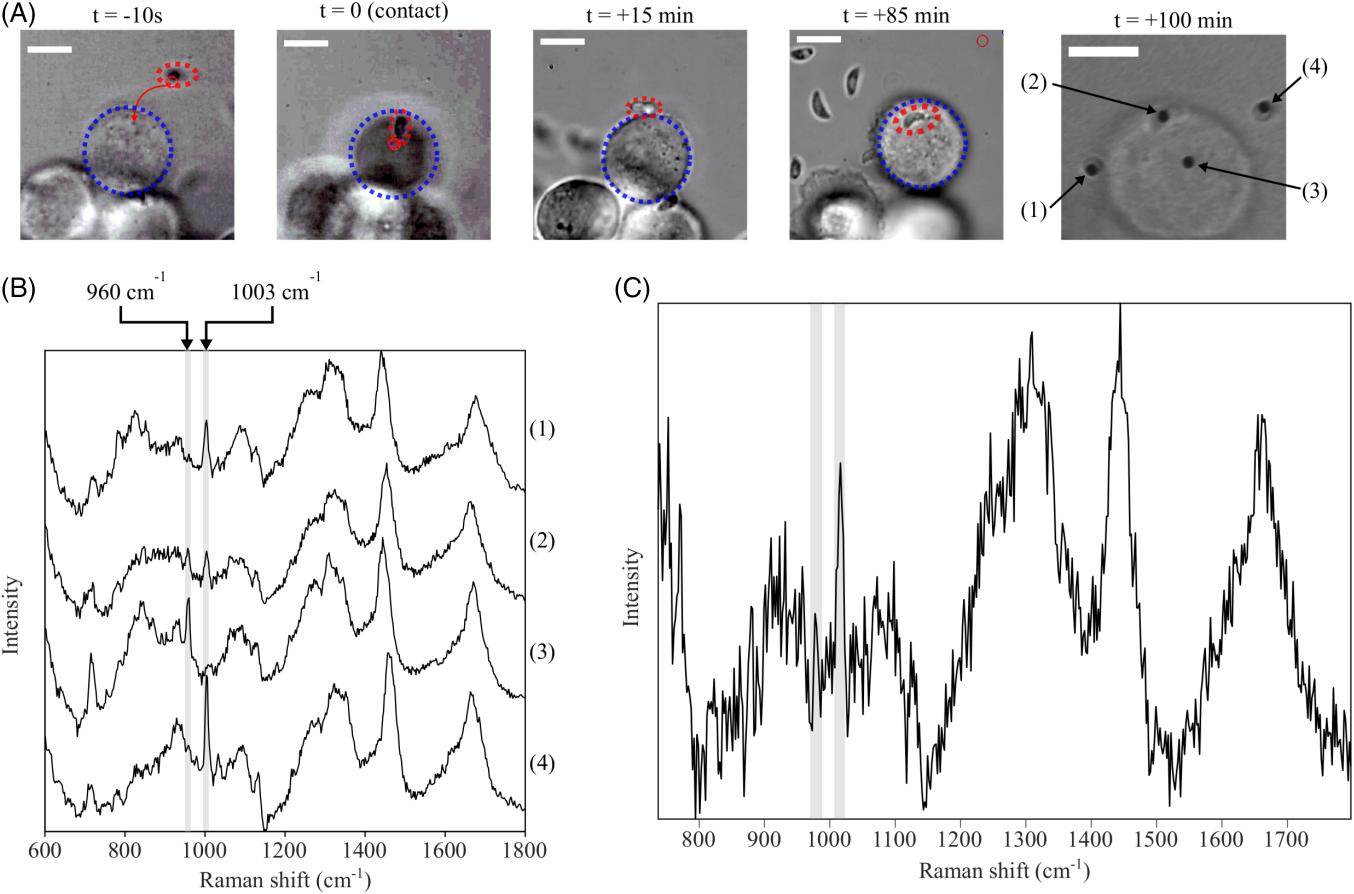
(A) Bright‐field microscope images of the early events of *T. gondii* infection of ECs initiated by optical trapping of a single parasite outlined by dashed red line (scale bars = 10 μm). (B) Simultaneous multipoint Raman spectra acquired 100 minutes after initiating host‐pathogen contact of external optically trapped *T. gondii* (1) and (4), internalized *T. gondii* (2), and EC cytoplasm (3) (acquisition time 60 seconds for simultaneously acquired spectra). (C) The calculated spectrum of the internalized *T. gondii* after subtraction of the EC contribution

The HOT‐RMS instrument used here is described fully in reference 19. Briefly, a CW Ti:Sapphire laser at 785 nm with approximately 1 W output power is expanded onto a phase‐only liquid‐crystal spatial light modulator (LC‐SLM), which is used to structure the beam to form the optical traps at the sample, with real‐time interactive update using RedTweezers software developed by Bowman et al.^21^ The beam is relayed via a telescope to the back aperture of a 60× 1.2 NA water immersion microscope objective (Olympus, UPLSAPO, Tokyo, Japan), placed on an incubated (Solent Scientific, Portsmouth, UK) inverted microscope (Olympus IX71). Typically six separate focused laser spots were generated at the sample, with an estimated 50 mW per beam (using method outlined in reference^19^). Raman scattering excited from the same laser used for optical trapping is collected in the backward direction, passed through a dichroic filter and focused onto a digital micromirror device (DMD) by a 25 mm diameter, 200 mm lens (Thorlabs AC254‐200‐B, Newton, New Jersey), resulting in a magnification from sample to DMD plane of 200/18 = 11.1×. The DMD is utilized as a reflective software‐controlled reconfigurable pinhole mask, allowing confocal Raman measurement from each optical trap to be carried out. Circular DMD pinholes of roughly 110 μm diameter were utilized, corresponding to approximately 15 DMD mirror pixels across. The Raman photons reflected from the DMD pinholes pass through a notch filter to remove residual laser light and focused into an imaging spectrometer (Princeton Instruments Acton LS785, Acton, Massachusetts) with thermoelectrically cooled CCD camera (Andor iDus, Belfast, UK). Acquisition times ranged from 2 seconds per Raman spectrum in Figure 1, 30 seconds for six parallel Raman spectra in Figure 2, and 60 seconds for four parallel Raman spectra in Figure 3. The multiple Raman spectra were acquired as a CCD image and processed in home‐built MATLAB code as described previously.^19^ Raman spectra shown in all figures (except for Figure 1C, which shows raw data) have had a polynomial baseline subtracted.

### 2.2 Maintenance of cell culture

Human brain microvascular endothelial cells (ECs) were used at passage 12 and were grown in Dulbecco's Modified Eagle Medium (DMEM) supplemented with 10% heat inactivated fetal bovine serum (FBS), 2 mM L‐glutamine, 1 mM Sodium Pyruvate, 1% MEM nonessential amino acids, 1% MEM vitamins and 2% penicillin/streptomycin as previously described.^22^ Cell culture was maintained in a monolayer in 75cm^2^ culture flasks inside an incubator in a humidified 5% CO2 −95% air at 37°C. Cells were considered confluent when their expansion had reached a point where cells touched each other on all sides and no intercellular gaps were observed. When cells reached at least 80% confluency (~3‐5 days), cell monolayers were trypsinized using trypsin‐EDTA (Invitrogen, GIBCO, Carlsbad, California,) and cells in suspension were seeded at a 1:3 passage ratio in new flasks. To rule out if cell viability could be regarded as a factor affecting host cell‐parasite interactions, which in turn might affect subsequent measurements, viability of ECs was assessed on a minimum of 100 cells using trypan blue exclusion assay prior to inoculation onto culture chambers or flasks. Only cells with >98% viability were used.

### 2.3 Propagation and purification of *T. gondii* tachyzoites

Tachyzoites of *T. gondii* genotype I (RH strain) were maintained by passage in EC cultures grown in DMEM using the same culture conditions described above. The culture flask infected with *T. gondii* was observed daily under inverted microscope. When >80% of the cell monolayers were destroyed, cell scrapper was used to detach the remaining attached cells in the flask. Detached cells and media were collected into a 50‐mL tube and centrifuged at 3000×*g* for 5 minutes. Supernatant was discarded and the pellet was resuspend in the medium. The tachyzoites were purified from their feeder cell cultures by passage through PD‐10 Desalting columns packed with Sephadex G‐25.^23^ The purified parasites were centrifuged at 800×*g*, resuspended in fresh culture medium, and counted using a Neubauer‐improved counting chamber (Marienfeld‐Superior) and Leica DM 1 L inverted microscope (Leica Microsystems, Wetzlar, Germany).

### 2.4 Infection protocol and measurement procedure

ECs (104 cells/mL) were seeded in titanium cell‐chambers, which are fitted in six‐well plastic cell culture plates, with a volume of 2 mL DMEM/chamber. The titanium cell chambers were custom‐built with fused quartz coverslips (0.17 mm thick) at the bottom to enable acquisition of Raman spectra. Cells were incubated overnight at 37°C in a humidified atmosphere of 5% CO2 −95% air. Then, cell monolayer was starved of serum for 18 hours, followed by incubation in L‐Phe(D8)‐supplemented DMEM for 7 days to ensure that cells were uniformly saturated and that L‐Phe has been fully substituted by L‐Phe(D8). Prior to the addition of *T. gondii* tachyzoites to the cells, the culture medium was replaced with L‐Phe‐free media to avoid any false labeling of the parasite with L‐Phe(D8) while in the extracellular medium before cell invasion. Parasites are grown in a medium containing L‐Phe, but they were transferred to L‐Phe‐free media before addition to the cells and during host cell invasion. This was performed to avoid introducing L‐Phe to L‐Phe(D8)‐labeled cells and ensure that the only source of Phe to the tachyzoites is from the L‐Phe(D8)‐labeled ECs. Infection was initiated by adding *T. gondii* tachyzoites at a host‐parasite ratio of 1:2 (ie, MOI; multiplicity of infection of 2) in 2‐mL fresh L‐Phe(D8)‐free DMEM. The control (uninfected) chambers received only 2‐mL DMEM. Culture plates were then incubated to allow infection to progress within ECs. A total of 6 coverslip samples were measured on separate days, with typically 3‐6 regions monitored by bright‐field imaging after manipulation of 5‐10 *T. gondii* parasites toward ECs using the optical traps. Infection efficiency of the manipulated parasites was observed to be <10%, though the natural infection process was observed to progress after Raman measurements were carried out.

## 3 RESULTS AND DISCUSSION

### 3.1 Viability of optically trapped *T. gondii*

First, we utilized the HOT‐Raman instrument for trapping and manipulation of individual *T. gondii* tachyzoites while enabling acquisition of Raman spectra without compromising host cell viability. Figure 1A shows representative examples of Raman spectra of individual optically trapped *T. gondii* tachyzoites, indicating the Raman band at 1003 cm^−1^ assigned to the breathing mode of _L_‐Phe, the focus of this study. The other Raman bands have been extensively reported in the literature (reviewed in^8^), and can be assigned to molecular vibrational modes of proteins, lipids, nucleic acids and carbohydrates. For comparison, Figure 1B shows a typical Raman spectra acquired from human ECs grown in culture medium supplemented with deuterated Phe (_L_‐Phe(D8)), confirming the shifting of the 1003 cm^−1^ band to 960 cm^−1^. All other bands assigned to endogenous molecules are unchanged. Time‐course spectra of optically trapped *T. gondii* (Figure 1C) were acquired over a duration of 4 minutes to investigate potential spectral changes caused by adverse effects caused by the laser irradiation. Although the 4 minute duration was much longer than the acquisition time of Raman spectra (2 seconds), the results in Figure 1C show that apart from minor drifts in baseline, no significant spectral changes were observed. In one case (out of seven), a step change in the baseline was observed, likely caused by the realignment of the *T. gondii* in the optical trap. However, this change in baseline was not accompanied by any changes in the Raman bands.

### 3.2 Estimation of spectrum contribution from host and parasite cells

Figure 2A presents Raman spectra of a typical EC infected with a single *T. gondii* tachyzoite (approximately 15 minutes post‐infection). Figure 2A includes spectra acquired at the location corresponding to the *T. gondii*, as well as at locations corresponding to nearby EC cytoplasm. While the spectra of EC cytoplasm show only the 960 cm^−1^ band assigned to _L_‐Phe(D), the spectrum obtained by focusing the laser on the *T. gondii* contains both the 960 cm^−1^ and 1003 cm^−1^, assigned to native _L_‐Phe and _L_‐Phe(D), respectively. Given that the size of the *T. gondii* is typically 1.5 μm thickness and 6 μm length, Raman scattering will be excited from a sampling volume extending in the EC cytoplasm. Therefore, in order to establish whether the measured 960 cm^−1^ Raman band can be used as an indication of _L_‐Phe(D) uptake by *T. gondii* from the host EC, subtraction of the EC contribution was required. The contribution of the *T. gondii* and EC to the measured Raman spectrum was expressed by the weightings *c*_1_ and *c*_2_, with *c*_1_ + *c*_2_ = 1. Then the Raman spectrum of the *T. gondii* was calculated using:

(1) $$S_{\mathrm{TG}}=\frac{S_{m}-c_{2}∙S_{\mathrm{EC}}}{c_{1}},$$

where *S*_*m*_ and *S*_EC_ are the spectra measured at the location of *T. gondii* and the cytoplasm, respectively, presented in Figure 2A.

In general, the weightings *c*_1_and *c*_2_ depend on the three‐dimensional (3D) profiles of the point spread functions corresponding to the illumination (defined by excitation laser irradiance) and detection configuration of the confocal Raman spectrometer. Using the numerical aperture of the microscope objective NA = *n*sin*θ* = 1.2 (*n* = 1.33 is refractive index of water), the waist of the laser beam exciting the Raman scattering (TEM_00_ mode of the Ti:sapphire laser) was calculated using:

(2) $$w_{0}=\frac{\lambda }{\pi \;n\tan\theta \;}=220\;\mathrm{nm}.$$

Given that the beam waist is significantly smaller than the transversal size of *T. gondii*, the sampling volume was estimated using a simplified model considering only the axial distribution of the laser irradiation and detection point spread function:

(3) $$\mathrm{PSF}\left(z\right)={\mathrm{PSF}}_{L}\left(z\right)\times \mathrm{PS}F_{D}\left(z\right),$$

where indices *L* and *D* refer to the laser illumination and detection system, respectively. The laser illumination point spread function PSF_*L*_(*z*) is defined by the laser irradiance profile, which for a Gaussian beam is (Figure 2B):

(4) $${\mathrm{PSF}}_{L}\left(z\right)=I_{L}\left(z\right)=\frac{a}{\pi ∙w{\left(z\right)}^{2}},$$

where *a* is a normalization factor and

(5) $$w\left(z\right)=w_{0}\;\sqrt{1+{\left(\frac{z\lambda }{\pi \;w_{0}^{2}}\right)}^{2}.}$$

In general, for an infinitely small pinhole, the detection point spread function, PSF_*D*_(*z*), is considered identical to the laser illumination profile (if differences between excitation and detection wavelength are neglected). However, the low efficiency of Raman scattering requires the use of a larger pinhole, particularly when dealing with biological samples. In this study, the diameter of the pinhole was set to double the Airy disk diameter (2 A.U.), therefore the assumption that PSF_*D*_(*z*) = PSF_*L*_(*z*) is not no longer valid. Thus, the PSF_*D*_(*z*) we determined by measuring the z‐profile of the Raman intensity of a polystyrene bead (3 μm diameter) scanned axially through the focal plane. This was achieved by shifting the focus of the laser beam using the SLM without altering the detection system (Figure 2C). The PSF_*D*_(*z*) was then determined by deconvolution (Figure 2B) and was found to have a full‐width at the half maximum of 4.4 μm. The overall axial point spread function of the instrument calculated a PSF(*z*) = PSF_*L*_(*z*) × PSF_*D*_(*z*) is presented in Figure 2C, and has a full‐width at the half maximum of 1.8 μm. This value is in good agreement with the empirical estimates of 2.5 λ = 2 μm based on Wilson et al.^24^ Using the model in Figure 2C, the weighting *c*_1_ describing the contribution of the *T. gondii* to the measured Raman spectrum was calculated using:

(6) $$c_{1}=\frac{\int_{-r}^{r}\mathrm{PSF}\left(z\right)\mathrm{dz}}{\int_{-D}^{D}\mathrm{PSF}\left(z\right)\mathrm{dz}},$$

where *r* and *R* are the radii of *T. gondii* and EC, respectively, and $D=\sqrt{R^{2}-{\left(R-r\right)}^{2}}$. In this case, the size of *T. gondii* and EC was obtained from the images captured with the microscope camera (*r* = 1.0 ± 0.2 μm and *R* = 8.5 ± 0.2 μm), yielding a value for *c*_1_ = 0.51 ± 0.06.

The calculated spectrum of *T. gondii* (*S*_TG_) is presented in Figure 2D. For subtraction, the Raman spectrum at the location in the EC cytoplasm closest to *T. gondii* (highlighted by the symbol *) was used as *S*_EC_. The result indicates that the calculated spectrum of *T. gondii* (*S*_TG_) does not have a band at 960 cm^−1^, which indicates that no uptake of _L_‐Phe(D) from the host EC has occurred.

After calibration of the sampling volume, the HOT‐Raman was used to induce interaction of *T. gondii* and ECs with control of timing and interaction between cells at early stage of infection. Simultaneous and in situ measurements are important because the molecular events that shape the parasite‐host cell interaction changes in time scales from minutes to hours or days.

### 3.3 Initiation and measurement of early stage of a host‐pathogen infection

The video frames shown in Figure 3A illustrate the manipulation of *T. gondii* parasites toward an adherent EC on the quartz coverslip surface. Multiple isolated *T. gondii* tachyzoites were trapped and brought into contact with a EC cell membrane surface, with typically fewer than 10% appearing to attach after several minutes of observation. After initial manipulation of the parasites toward the ECs, the laser is blocked from the sample to allow the interaction to progress naturally from this point and avoid unnecessary laser exposure. In the first part of a successful attachment, the apical end of *T. gondii* appears fixed at the cell membrane surface of the EC close to the position it was manipulated toward at *t* = 0 using the optical traps, as can be observed in the frame at *t* = +15 minutes. At this point, the parasite appears able to be partially trapped, while tethered at one end to the host cell. At *t* = +85 minutes, the parasite has entered the cytoplasm of the host cell and is no longer able to be moved by the optical trap.

*Toxoplasma gondii* tachyzoites which had become internalized were distinguishable from those simply remained extracellular (ie, above/below the cell) by their motion (as Brownian motion was not observed for internalized parasites living inside the parasitophorous vacuole) and the relative focus of the cytoplasm and the parasite from microscope images. At *t* = 100 minutes, Raman spectra were acquired from four different locations within the host‐pathogen system simultaneously, as can be observed in Figure 2B. The spectra labeled (1)‐(4) correspond to (1): a newly attached partially trapped parasite in the first stage of invasion (similar to that seen at *t* = +15 minutes in Figure 3A), (2): the internalized parasite manipulated toward the ECs at *t* = 0, (3): the cytoplasm of the EC (used for subtraction), and (4): an isolated *T. gondii* tachyzoites trapped away from the ECs. It can be observed that the isolated parasite (4) and the attached parasite at the early stage of invasion (1) exhibit the standard 1004 cm^−1^ phenyl Raman band, with no observable deuterated phenylalanine (_L_‐Phe(D8)) peak at 960 cm^−1^ in either spectrum. In contrast, the Raman spectrum from the cytoplasm of the ECs (3) shows only the presence of the deuterated phenylalanine Raman band at 960 cm^−1^, as expected.

The Raman spectrum of the internalized *T. gondii* (2) appears to contain Raman bands at both 960 and 1004 cm^−1^. Using the sizes of *T. gondii* (*r* = 1.0 ± 0.2 μm) and EC (*R* = 9.5 ± 0.2 μm) obtained from the camera images, the value of the weightings describing the contributions of the *T. gondii* and EC were *c*_1_ = 0.51 and *c*_2_ = 0.49. The spectrum of *T. gondii* after subtracting the EC contribution (spectrum in the cytoplasm was used as *S*_EC_) (Figure 3C) contains the 960 cm^−1^ band indicating that even at this early stage (15 minutes after entering the EC), *T. gondii* has acquired a detectable amount of _L_‐Phe(D8) from the host cell. Based on the corrected spectrum, the ratio of the band areas was *I*_*960*_ */I*_*1004*_ = 0.25 ± 0.10, suggesting that a significant amount of the Phe in *T. gondii* was acquired from the host ECs.

## 4 CONCLUSION

We utilized an integrated HOT and RMS based time‐lapse imaging system for characterization of the parasite *T. gondii* interaction with the human brain microvascular endothelial cells. HOT‐RMS allowed the initiation and direct visualization of the dynamic interactions between *T. gondii* and host cells, and enabled the detection of trafficking of the isotope‐labeled amino acid L‐Phe(D8) between host cell and the parasite. HOT‐RMS system was capable of distinguishing the L‐Phe(D8) of the host cells from the parasite, while simultaneously achieving control over the induction of the interaction between the parasites and the host cells; this capability, not achievable by any other conventional approach, is particularly useful because living systems are highly dynamic and often exhibit nonlinear behavior. While these preliminary results used L‐Phe(D) as an example of the molecular exchange, experiments could be expanded to other molecules based on SILAC or other small molecular tags. Furthermore, automation and combination with microfluidic technology for control of initial cell populations across the sample would enable these measurements to be carried out with higher accuracy and throughput, enabling more statistically significant observations of single‐cell infection events to be carried out. It is anticipated that this novel research approach will allow a better and clearer determination of the specific environments and conditions that facilitate parasite invasion and the molecular determinants that mediate the host‐parasite interaction.

## CONFLICT OF INTEREST

The authors declare no financial or commercial conflict of interest.
